# Comparison of Multiobjective Evolutionary Algorithms for Operations Scheduling under Machine Availability Constraints

**DOI:** 10.1155/2013/418396

**Published:** 2013-12-30

**Authors:** M. Frutos, M. Méndez, F. Tohmé, D. Broz

**Affiliations:** ^1^Department of Engineering and Instituto de Investigaciones Económicas y Sociales del Sur (IIESS-CONICET), Universidad Nacional del Sur, Avenida. Alem 1253, 8000 Bahía Blanca, Argentina; ^2^Instituto Universitario de Sistemas Inteligentes y Aplicaciones Numéricas en Ingeniería (SIANI), Universidad de Las Palmas de Gran Canaria (ULPGC), Campus Universitario de Tafira, 35017 Las Palmas de Gran Canaria, Spain; ^3^Department of Economics and Instituto de Investigaciones Económicas y Sociales del Sur (IIESS-CONICET), Universidad Nacional del Sur (UNS), 12 de Octubre 1198, 8000 Bahía Blanca, Argentina

## Abstract

Many of the problems that arise in production systems can be handled with multiobjective techniques. One of those problems is that of scheduling operations subject to constraints on the availability of machines and buffer capacity. In this paper we analyze different Evolutionary multiobjective Algorithms (MOEAs) for this kind of problems. We consider an experimental framework in which we schedule production operations for four real world Job-Shop contexts using three algorithms, NSGAII, SPEA2, and IBEA. Using two performance indexes, Hypervolume and R2, we found that SPEA2 and IBEA are the most efficient for the tasks at hand. On the other hand IBEA seems to be a better choice of tool since it yields more solutions in the approximate Pareto frontier.

## 1. Introduction

Production planning problems involve the allocation of scarce resources to different tasks in such way as to optimize one or more efficiency-related goals [1]. In most cases, these problems are analyzed as instances of the Job-Shop Scheduling Problem, in which given a set of machines and a list of jobs, represented as ordered sequences of operations, to be run on the machines, the goal is to minimize, in particular, the processing time of all jobs, known as *makespan* [2]. This problem belongs to the class NP-hard [3], for which no efficient algorithms are known to run in reasonable execution times. The literature focuses mostly on single-objective versions of the problem, despite the fact that several authors have stated that a genuine scheduling problem involves more than one objective when production efficiency is sought [4]. If this is indeed the appropriate approach, Multiobjective Evolutionary Algorithms (MOEAs) are the tools of choice [5, 6]. Their main advantages are their ease of adaptation to different instances and their overall efficiency. Only the fitness function has to be known, instead of its rates of variation, making the evolutionary algorithms efficient for problems that cannot be solved in reasonable time with gradient-based methods.

In this paper we will evaluate the performance of three MOEAs on Job-Shop scheduling problems [7, 8]: NSGAII [9], SPEA2 [10], and IBEA [11]. These competing approaches all use domination and elitism to reach the best possible approximation to the solutions of multiobjective problems. They differ in the strategies on which they are based (lack of a predefined sharing parameter, in the case of NSGAII; a fine grained fitness assignment procedure in SPEA2; a qualitative indicator function over Pareto approximations in IBEA), but their performance on different instances tends to be the best available in the literature. We, furthermore, add extra constraints, on both the availability of machines and the buffer capacity. We run the algorithms on real-world problems in which nonstandardized production (like in the Job-Shop context) has to share machines with standardized processes that have priority over the former.

## 2. Job-Shop Scheduling Problem

The Job-Shop Scheduling Problem is quite complex. It has been analytically solved for 1, 2, and 3 machines and a small number of jobs. Only a few efficient algorithms have been found for 4 or more machines and 3 or more jobs. This is due to the combinatorial explosion of possible schedules. In the next subsections we will review the state of the art in this matter and formally define the problem, introducing the objectives to be optimized in our analysis.

### 2.1. State of the Art

A brief review of the approaches to the Job-Shop Scheduling Problem shows a multiplicity of techniques. So, for instance, [12] presents a tabu search method intended to minimize total tardiness, while [13] presents a GRASP (Greedy Randomized Adaptive Search Procedures) algorithm that minimizes the makespan, and [14] uses a HACO (Hybrid Ant Colony Optimization) algorithm for the same goal. In [15] a localization approach is suggested, minimizing both makespan and the machine load. In [16] a mathematical model is introduced, able to solve only small instances of the problem. Closer to our object of study, [17] presents a hybrid algorithm also minimizing makespan, while [7] introduces a genetic algorithm in which the representation makes every schedule feasible. In [18] a genetic algorithm is presented for which the control parameters have been tuned to optimize makespan. In [19] dispatch rules are proposed and at each generation the search space is explored by means of schemes, again with the objective of minimizing makespan. This approach is generalized in [20] where an architecture based on an evolutionary algorithm is combined with learning through schemes and the generation of populations by means of combined dispatch rules. In [21] a multiple scenarios genetic algorithm is introduced, in which each scenario corresponds to an operation and each feasible machine to a state. In [22] a genetic algorithm profits from the localization approach presented in [15]. A class of mutations consists in allocating operations from machines with heavy loads to less loaded ones. In [2] a hybrid genetic algorithm solves the problem with the proviso that no waiting time is allowed among operations for any job, minimizing the makespan. Finally, [23] presents an evolutionary algorithm minimizing the makespan, the total work load, and the maximum load.

### 2.2. Formal Definitions

The Job-Shop Scheduling problem consists in finding an optimal allocation of a class of n jobs *J* = {*J*_*j*_}_*j*=1_^*n*^, to be processed by a set of m machines *M* = {*M*_*k*_}_*k*=1_^*m*^. Each job is described as a sequence of tasks that can be performed in sequence *J*_*j*_ ≡ *S*_1_, ..., *S*_*nj*_. The operation of a task *S*_*i*_ of job *J*_*j*_ on machine *M*_*k*_ is denoted as *O*_*ij*_^*k*^. Operation *O*_*ij*_^*k*^ requires using machine *M*_*k*_ for an uninterrupted *processing time* *τ*_*ij*_^*k*^. A solution for this problem involves the determination of the starting time *t*_*ij*_^*k*^ of each operation *O*_*ij*_^*k*^ while optimizing the objectives [8]. Here we consider three objectives. The first one is the minimization of the *makespan* (1). This involves shortening the total time required for the *n* jobs. The second objective is the minimization of the *mean flow time* (2). This amounts to reducing the number of jobs processed in parallel. Finally, we seek to minimize the effects on the makespan of variations of the *τ*_*ij*_^*k*^, for each *O*_*ij*_^*k*^. For this we run microsimulations to find the *variance* of the first objective *σ* ^2^(*C*_max⁡_^*j*^) [24]. The minimization of this variance ensures the stability of solutions (3):

(1) $$\begin{array}{c} f_{1}:\min C_{\max}^{j}=\underset{i\in S_{j}}{\sum }\underset{k\in M}{\max}\left(t_{ij}^{k}+\tau_{ij}^{k}\right), \end{array}$$

(2) $$\begin{array}{c} f_{2}:\min\bar{F}=\frac{1}{n}\underset{j\in J}{\sum }\;\underset{i=m}{\sum }\left(t_{ij}^{h}+\tau_{ij}^{h}-t_{1j}^{k}\right), \end{array}$$

(3) $$\begin{array}{c} f_{3}:\min\sigma^{2}\left(C_{\max}^{j}\right). \end{array}$$

We assume the nonnegativity of the starting time *t*_*ij*_^*k*^ of each *O*_*ij*_^*k*^: *t*_*ij*_^*k*^ ≥ 0. Besides, we have (joint) precedence constraints of operations for each job: if *i* ≥ *s*, *S*_*i*_, *S*_*s*_ are tasks of *J*_*j*_ and *S*_*i*_ is executed on *M*_*k*_, while *S*_*s*_ on *M*_*h*_, then *t*_*ij*_^*k*^ − *t*_*sj*_^*h*^ ≥ *τ*_*sj*_^*h*^. Finally, the (disjoint) nonjuxtaposition constraints are applied on each machine: if *S*_*i*_ is a task in *J*_*j*_ while *S*_*s*_ in *J*_*p*_, both to be executed on *M*_*k*_, we have that *t*_*ij*_^*k*^ − *t*_*sp*_^*k*^ ≥ *τ*_*sp*_^*k*^. The purpose of the latter constraints is to warrant that no machine carries out two operations at the same time. Two additional constraints involve the availability of machines and the capacity of the buffer. The first ones limit the operational interval of each machine; that is, (*t*_*ij*_^*k*^, *t*_*ij*_^*k*^ + *τ*_*ij*_^*k*^) must be larger than the operational interval corresponding to the standardized operational interval of machine *M*_*k*_. The second group of constraints limits the number of operations *O*_*ij*_^*k*^ on machine *M*_*k*_ that are on a waiting list. The buffer can either hold 0 operations (*no-wait*) or *n* − 1 operations (*nonrestricted*).

## 3. Evolutionary Multiobjective Algorithms

Evolutionary algorithms imitate genetic processes by improving solutions, pairing existing solutions as if they were DNA chains, and creating new chains. A *chromosome* is composed by smaller units called *genes*. For our problem the chromosomes identify a schedule of operations. Evolutionary improvements should end up yielding the optimal schedule. To show how this works we use an example with three jobs and three machines (3 × 3). The total problem involves nine operations. Their corresponding processing times (*τ*_*ij*_^*k*^) and variances (*σ*_*ij*_^*k*^ ^2^) for the machine in which they run (*M*_*k*_) are shown in Table 1.

### 3.1. The Evolutionary Phase

**Figure 1:**
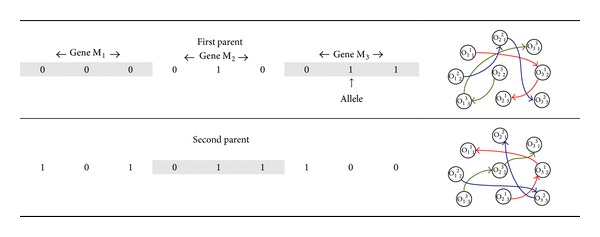
Chromosomes representing schedules.

To represent an individual schedule, we use the notation proposed in [8]. The chromosome contains binary variables and the chain has as many genes as machines in the problem. Each gene has a certain number of *alleles*, depending on the number of jobs of the problem. More precisely, the size of a gene is ⌈log⁡(*n*!)/log⁡(2)⌉, while the total size of the chromosome is *m*∗⌈log⁡(*n*!)/log⁡(2)⌉. For each gene the sequence of binary numbers represents the sequence of jobs in the corresponding machine *M*_*k*_. In our example we choose 000 → 1 | 2 | 3, 001 → 1 | 3 | 2, 010 → 2 | 1 | 3, 011 → 2 | 3 | 1, 100 → 3 | 1 | 2 and 101 → 3 | 2 | 1. Consider the first parent in Figure 1. The first gene, of machine *M*_1_, is 000. This means that the sequence of jobs in the machine is 1, 2, and 3. For *M*_2_ the gene is 010, that is, the sequence of jobs, is 2, 1, and 3. Finally, for *M*_3_ the gene is 011, and therefore the jobs are 2, 3, and 1.

**Figure 2:**
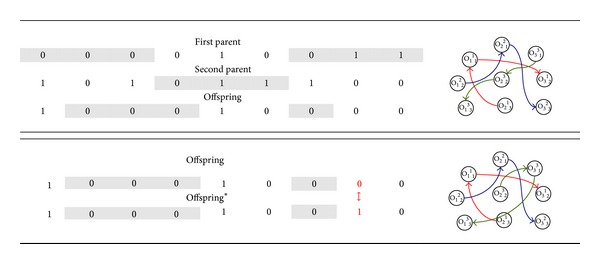
Crossover and mutation.

A crossover operator acts on pairs of chromosomes. It aligns the chromosomes, cuts them at a certain points, and exchanges the fragments between the chromosomes. To see how it works, consider again Figure 1 the two “parents” to crossover, called First Parent and Second Parent. A “child” is built incorporating randomly elements from both parents (the offspring in Figure 2). The other child is obtained by inverting the choices made for the other one. This crossover, called uniform, yields better results exploring solutions close to the Pareto frontier. A mutation varies the binary values of one or more alleles of the gene. This variation is applied at random points of the chromosome, generating an individual with small differences with the original chromosome. In our case 10% of the alleles of the chromosomes are changed. In Figure 2, offspring* represents the mutated chromosome.

### 3.2. Selection of MOEAs

We consider three Multiobjective Evolutionary Algorithms: Nondominated Sorting Genetic Algorithm II (NSGAII) [9], Strength Pareto Evolutionary Algorithm 2 (SPEA2) [10], and Indicator-Based Evolutionary Algorithm (IBEA) [11]. They have been applied in the literature to engineering problems. It classifies the population in fronts. Each individual is assigned a rank corresponding to its nondominance level. This method ensures that the best solutions will remain at the next iteration. Elitism is therefore already incorporated without requiring an external procedure. NSGAII further reduces the complexity of the ordering procedure, based on nondominance, of its predecessor NSGA and allows the preservation of diversity by means of a technique called *crowding*. SPEA2 is a variant of SPEA. It assigns fitness by considering for each individual the class of individuals that dominate it and the class of those that are dominated by the individual. SPEA2 uses also a “closest neighbor” technique that values the density to improve the search. Finally, IBEA incorporates indexes of multiobjective quality, providing an alternative to Pareto dominance as a guide in the search.

## 4. Implementation and Design of Experiments

For our experiments we used four instances drawn from real cases: C_1 (15 × 20 problem with 157 operations), C_2 (20 × 20, 242 operations), C_3 (20 × 25, 412 operations), and C_4 (25 × 25, 597 operations). For each one we took into account the characteristics of the buffer, namely, no-wait and nonrestricted. Once the appropriate number of generations for the evolutionary phase and the production configuration for the microsimulations are defined, we run the experiments using PISA (A Platform and Programming Language Independent Interface for Search Algorithms) [25]. The parameters and characteristics of the experiments are shown in Table 2. For IBEA we chose the *additive epsilon* index. The other parameters keep their PISA predefined values. For each problem, the algorithm was run 30 times. From the class of solutions obtained, the dominated ones were eliminated. The running time of problems C_1, C_2, and C_3 was less than 30 minutes (Processor: 2.1 GHz AMD Turion X2 Ultra Dual-Core, Memory: 4 GB 800 MHz DDR2). C_4, instead, took in average 85,71 minutes.

## 5. Results

We provide in the next subsections analyses of the Pareto frontiers and a comparison of the three algorithms by means of the Hypervolume and R2 indexes.

### 5.1. Pareto Frontiers

**Figure 3:**
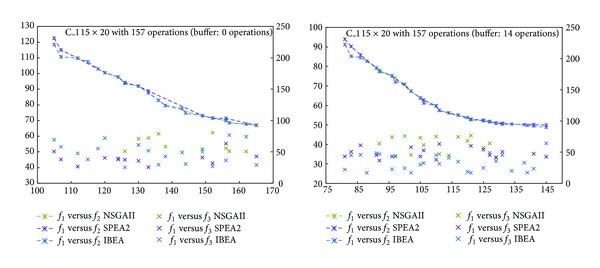
Pareto frontiers, *f*_1_ versus *f*_2_ and *f*_1_ versus *f*_3_, C_1.

**Figure 4:**
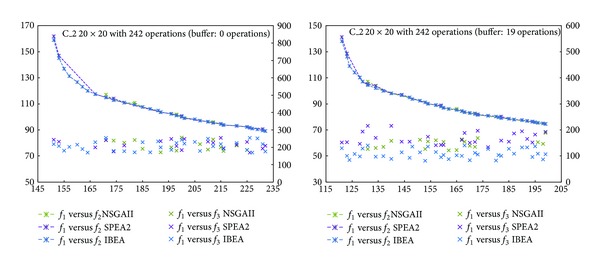
Pareto frontiers, *f*_1_ versus *f*_2_ and *f*_1_ versus *f*_3_, C_2.

**Figure 5:**
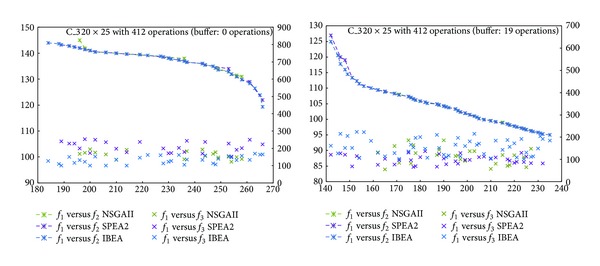
Pareto frontiers, *f*_1_ versus *f*_2_ and *f*_1_ versus *f*_3_, C_3.

**Figure 6:**
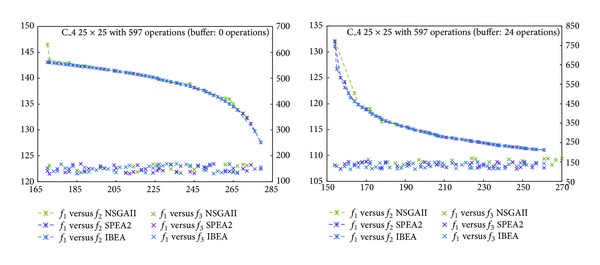
Pareto frontiers, *f*_1_ versus *f*_2_ and *f*_1_ versus *f*_3_, C_4.

The frontiers obtained in our experiments are shown in Figures 3 to 6. The horizontal axis represents objective *f*_1_, the left vertical one, *f*_2_, while the right vertical, *f*_3_. Comparing *f*_1_ and *f*_2_, the fronts of the three algorithms look alike, although IBEA generated a better distributed front. NSGAII, instead, generated an incomplete frontier. With respect to *f*_1_ versus *f*_3_, we see that for C_1 with a buffer of 0 operations, algorithms SPEA2 and IBEA obtained better values than NSGAII (Figure 3). For C_1 with a buffer of 14 operations, IBEA got the best variance values (Figure 3). For C_2 the 0 operations buffer makes no difference (Figure 4). For C_2 with a buffer of 19 operations, IBEA yielded the best values in variance (Figure 4). On C_3 with a 0 operations buffer, NSGAII and IBEA got better values than SPEA2 (Figure 5), while for the 19 operations buffer, NSGAII and SPEA2 obtained better values than IBEA (Figure 5). Finally, on C_4, a 0 or 24 operations buffer made no difference (Figure 6).

### 5.2. Comparison through Quality Indexes

**Figure 7:**
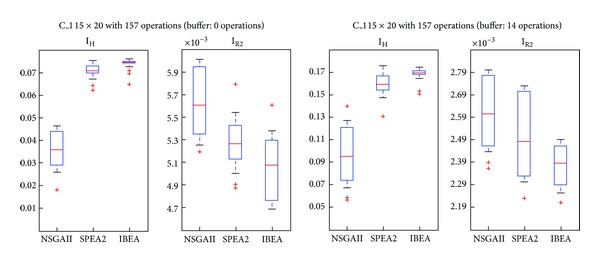
Boxplot/values for NSGAII, SPEA2, and IBEA, C_1.

**Figure 8:**
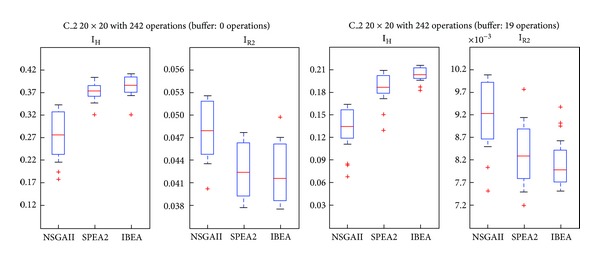
Boxplot/values for NSGAII, SPEA2, and IBEA, C_2.

**Figure 9:**
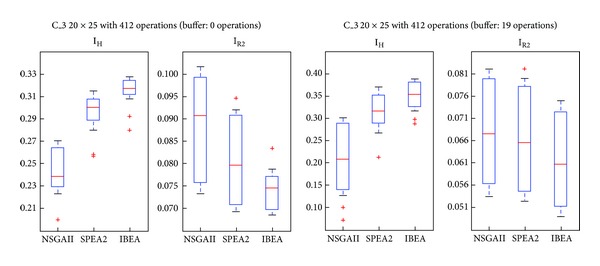
Boxplot/values for NSGAII, SPEA2, and IBEA, C_3.

**Figure 10:**
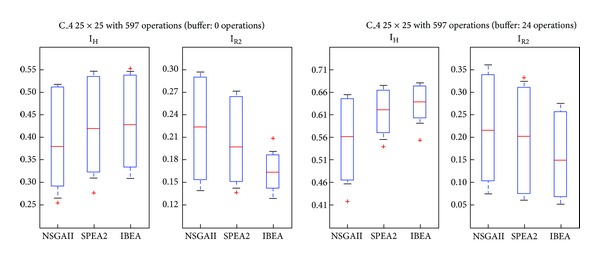
Boxplot/values for NSGAII, SPEA2, and IBEA, C_4.

We compared the results according two indexes: Hypervolume [26] and R2 [27]. These are the usually recommended approaches to the evaluation of Pareto fronts. They provide slightly different advantages in the assessment of the frontiers. On one hand, Hypervolume seems fitter because it satisfies strong monotonicity while R2 only weak monotonicity. On the other, the former tends to be biased towards boundary solutions, while *R*2 is more uniform. Hypervolume requires a reference point to establish the area dominated by a given point, represented by the vector of its *f*_1_, *f*_2_, and *f*_2_ values. Thus, a higher index indicates that the algorithm yields better solutions. *R*2 estimates the degree of closeness of the solution to the real front. Therefore, a low index indicates that the algorithm yields better solutions. Figures 7 and 8 show cases in which SPEA2 and IBEA are better according both indexes.  Figure 9, instead, shows a case in which IBEA yields the better results, while Figure 10 presents a case in which there are no differences among the three algorithms. A possible explanation for NSGAII's general low degree of efficiency is that more than 2 objectives impair the crowding operator. Besides, it is well known that this algorithm is not efficient with binary representations.

To these casual observations we added a parametric statistical analysis, Fisher's test, with a significance level of 0.05. Problems C_1, C_2, and C_3 present significant differences in favor of SPEA2 and IBEA over NSGAII. Even if C_3 IBEA seems to perform better than SPEA2, the statistical analysis does not yield differences between these two algorithms. Finally, in C_4 there are no significant differences among the algorithms.  Table 3 shows the contribution of each algorithm to the approximate Pareto, formed by taking the nondominated solutions.

## 6. Conclusions

This paper presents an analysis of the performance of three different Multiobjective Evolutionary Algorithms in experiments with Job-Shop Scheduling Problems. It required the specification of parameters appropriate for the problems at hand, involving constraints on machine availability and buffer capacity. An important share of the running time of the algorithms corresponded to microsimulations of the variance of makespan of solutions. The comparison leads to the selection of SPEA2 and IBEA, while the contribution to the approximate Pareto frontier makes IBEA the most efficient algorithm for the problems at hand. Future work involves the extension of this comparison on other production environment problems. It seems also worthwhile analyzing the implications of the variance of makespan in comparison with other objectives.

## Figures and Tables

**Table 1:** Scheduling operations.

3 × 3 problem with 9 operations									
*J*_*j*_	*J*_1_			*J*_2_			*J*_3_		

*O*_*ij*_^*k*^	*O*_11_^1^	*O*_21_^2^	*O*_31_^3^	*O*_12_^2^	*O*_22_^3^	*O*_32_^1^	*O*_13_^3^	*O*_23_^1^	*O*_33_^2^

*τ*_*ij*_^*k*^	10	15	8	12	9	10	11	5	16
*σ*_*ij*_^*k*^ ^2^	4	4	2	4	3	5	2	1	4
*M*_*k*_	1	2	3	2	3	1	3	1	2

**Table 2:** Parameters and characteristics of the experiments.

	C_1 15 × 20, 157 op.	C_2 20 × 20, 242 op.	C_3 20 × 25, 412 op.	C_4 25 × 25, 597 op.
Inicialization	Random	Random	Random	Random
Representation	Binary	Binary	Binary	Binary
Number of genes	20	20	25	25
Size of gene	22	62	62	84
Size of chromosome	820	1240	1550	2100
Size of population	50	50	100	100
Generations	1000	1000	1000	1000
Crossover type	Uniform	Uniform	Uniform	Uniform
Probability of crossover	0.85	0.85	0.85	0.85
Mutation type	Alterate	Alterate	Alterate	Alterate
Mutated alleles	82	124	155	210
Probability of mutation	0.05	0.05	0.05	0.05
Number of objectives	3	3	3	3
Number of runs	30	30	30	30
Buffer limits	0–14	0–19	0–19	0–24

**Table 3:** Approximate Pareto frontier and the contribution of each algorithm.

Problem	NSGAII	SPEA2	IBEA
C_1 (no-wait)	50,00%	60,00%	100,00%
C_1 (non-restricted)	59,26%	66,67%	100,00%
C_2 (no-wait)	48,48%	60,61%	100,00%
C_2 (non-restricted)	51,28%	69,23%	100,00%
C_3 (no-wait)	52,94%	73,53%	100,00%
C_3 (non-restricted)	50,98%	74,51%	100,00%
C_4 (no-wait)	46,30%	77,78%	85,19%
C_4 (non-restricted)	56,14%	73,68%	91,23%
